# Nontuberculous mycobacteria by metagenomic next-generation sequencing: Three cases reports and literature review

**DOI:** 10.3389/fpubh.2022.972280

**Published:** 2022-11-14

**Authors:** Ying Liu, Xiaoxu Ma, Jiajun Chen, Huifen Wang, Zujiang Yu

**Affiliations:** ^1^Department of Infectious Diseases, The First Affiliated Hospital of Zhengzhou University, Zhengzhou, China; ^2^Department of Respiratory Diseases, The First Affiliated Hospital of Zhengzhou University, Zhengzhou, China; ^3^School of Public Health, Zhengzhou University, Zhengzhou, China; ^4^Gene Hospital of Henan Province, The First Affiliated Hospital of Zhengzhou University, Zhengzhou, China

**Keywords:** nontuberculous mycobacteria, nontuberculous mycobacterial lung disease, mNGS, diagnose, treatment

## Abstract

**Background:**

The increasing worldwide incidence of nontuberculous mycobacterial lung disease (NTM-LD) and the similarity of its manifestations to those of tuberculosis (TB) pose huge challenges in the diagnosis and treatment of NTM-LD, which is commonly misdiagnosed and mistreated as TB. Proper diagnosis and treatment at an early stage can greatly improve patient outcomes.

**Case presentation:**

*Mycobacterium avium* was identified by mNGS in lung tissue of case 1 and bronchioalveolar fluid from case 2 that was not identified using conventional microbiological methods. Multiple NTM species were detected in the blood mNGS samples from case 3 who had disseminated NTM infection. Although NTM was isolated from blood culture, conventional methods failed to identify the organisms to the level of species. All three patients were suffering from and being treated for myelodysplastic syndrome, rheumatoid arthritis, systemic lupus erythematosus, or acute lymphoblastic leukemia, making them immunosuppressed and susceptible to NTM infections. Case 1 and Case 2 significantly improved after anti-NTM treatment, but case 3 succumbed to the infection due to her underlying medical illness despite aggressive treatment.

**Conclusions:**

The cases in this study demonstrate the effectiveness of mNGS in facilitating and improving the clinical diagnosis of NTM infections. We propose combining mNGS with traditional diagnostic methods to identify pathogens at the early stages of the disease so that targeted treatment can be implemented.

## Introduction

Nontuberculous mycobacteria (NTM) cause infections in various tissues, with lung infections making up about 90% of these infections (1). To date, more than 190 NTM species and subspecies have been identified (2). The most common nontuberculous mycobacterial lung disease (NTM-LD) causing species are *Mycobacterium avium complex* (MAC), *Mycobacterium kansasii*, and *Mycobacterium abscessus*. NTM-LD presents mainly as fibrocavitary disease in patients with structural lung diseases, such as cystic fibrosis, chronic obstructive pulmonary disease, and bronchiectasis, and as nodular bronchiectatic disease of the lingula and middle lobe mainly seen in middle-aged and elderly women (3). NTM also causes infection in other tissue, including lymph and soft tissue (4, 5).

Metagenomic next-generation sequencing (mNGS) is a relatively new genomics-based technology with higher flux and faster sequencing speed compared with older sequencing technologies. mNGS offers unbiased pathogen detection by amplifying all nucleic acid sequences in samples with random primers (6). In this study, we used mNGS for early diagnosis of NTM-LD in the clinic.

## Materials and methods

### Sample preparation, DNA extraction, and library construction and sequencing

Bronchoalveolar lavage fluid (BALF), biopsy tissue, and blood samples were collected from patients according to the standard procedure. Blood samples were centrifuged at 1,500 rpm for 10 min at 4°C. The plasma layer was collected and transferred to a new tube and centrifuged at 12,000 rpm for 10 min at 4°C. Cell-free DNA (cfDNA) was extracted from the plasma using the TIANamp Micro DNA DP316 Kit (Tiangen Biotech, Beijing, China) according to the manufacturer's protocol. DNA was extracted from BALF and biopsy tissue samples using QIAamp^®^ UCP Pathogen Kit (Qiagen, Germany) in accordance with the manufacturer's instructions. A Qubit Fluorometer (Thermo Fisher Scientific, CA, USA) was used to quantify the extracted cfDNA and DNA samples.

The extracted cfDNA samples were used to construct DNA libraries utilizing the VAHTS Universal DNA Library Prep Kit V3 for Illumina^®^ (Vazyme, Nanjing, China). DNA libraries were constructed on the TruePrep DNA Library Prep Kit V2 for Illumina^®^ (Vazyme, Nanjing, China) using the DNA samples. The Agilent 2,100 Bioanalyzer (Agilent Technologies, Santa Clara, USA) was utilized for library quality control. These libraries were merged with others utilizing different index sequences and then sequenced using the single-ended 75 bp sequencing option on the Illumina NextSeq 550Dx platform. No-template control (NTC) samples (nuclease-free H_2_O) were used to monitor contaminations during each run.

### Bioinformatics analysis

Bcl2fastq software (v2.20.0.422, parameters used: –barcode-mismatches 0 –minimum-trimmed-read-length 50) was used to generate FASTQ files for each sample. Cutadapt V2.10 (-q 25, 25 -m 50) was used to remove adapter sequences and low-quality reads. High-quality reads were mapped to the human genome (hg38, https://hgdownload.soe.ucsc.edu/downloads.html#human) using default parameters on bwa-mem 2 V2.1, and then all unmapped reads were aligned to the NCBI nt database (https://ftp.ncbi.nlm.nih.gov/genomes/) using BLAST V2.9.0 + (-task megablast -num_alignments 10 -max_hsps 1 -evalue 1e-10) (7). Alignments were required to be full-length with at least 95% sequence identity. A customized Python script was used to identify species-specific alignments, and only alignments meeting the above criteria were used for further pathogen identification. The remaining microbes were defined as credible if the following criteria were met: (1) the microbe had at least 2 non-redundant mapped reads per million (RPM) raw sequence reads (except for NTM) and was not detected in corresponding NTC samples, or the RPM(sample)/RPM(NTC) was ≥ 5, which was our empirical cutoff for differentiating true-positive from background contamination; (2) due to the difficulty of detecting NTM, when at least one taxon-specific, high-quality aligned read was identified, the sample was reported as NTM positive.

### Case description

#### Case 1

**Figure 1:**
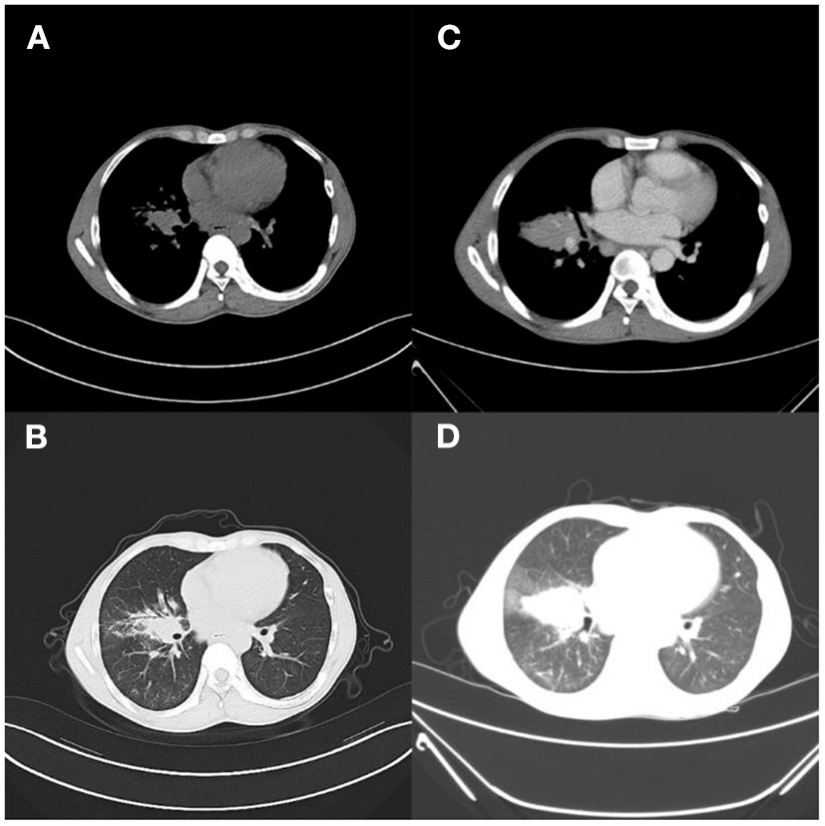
Case 1. **(A,B)** Outpatient lung CT; **(C,D)** 9 days after admission, pulmonary inflammation worsened, nodules increased, and lymph nodes were more enlarged.

A 35-year-old male was admitted to the hospital for fever accompanied by limb weakness, sweating, chills, and shivering for more than one month. He had a history of myelodysplastic syndrome, severe anemia, granulocytopenia, and chronic hepatitis B infection. The patient lost 1.5 kg since the onset of the fever and was treated with ibuprofen and amoxicillin, but when symptoms did not improve, he came to the outpatient department of our hospital for a lung CT which showed enlarged space occupying right hilum and mediastinal lymph node enlargement, left lower lung nodules, double pneumonia (Figure 1). Laboratory test abnormalities on the first day of admission included red blood cell count 2.44 × 10^12^ cells/L (reference range: 4.3–5.8), hemoglobin 73.0 g/L (reference range: 130–175), albumin 30.6g/L (reference range: 35–55), globulin 41.9g/L (reference range: 20–35), C-reactive protein 36.05mg/L (reference range: 0–10), erythrocyte sedimentation rate 93.00 mm/h (reference range: 0–15), and aspergillus galactomannan 5.23 ug/L (reference range: 0–0.85). Acid-fast staining (bronchoalveolar lavage fluid) was negative on day 3 of admission. Bacterial cultures were negative on both day 5 and day 26 of admission, and fungal cultures were negative on day 10. During hospitalization, recombinant human erythropoietin, recombinant human granulocyte stimulating factor, and intravenous human immunoglobulin were used to treat his myelodysplastic syndrome.

Levofloxacin, a broad-spectrum antibiotic, and rifampicin, an antituberculotic drug, were given empirically on the day of admission. But these days the patient has repeated fever, even sustained high fever. The specific cause of the patient's infection could not be determined because bacterial smear did not reveal pathogenic bacteria, and blood cultures were negative. Based on clinical experience, we used voriconazole against fungal infections, biapenem and doxycycline against bacterial infections, and glucocorticoids against anti-inflammatory responses (**Figure 6A**). One week after admission, a bronchoscopy biopsy showed pulmonary granulomatous lesions. Nine days after admission, the patient's chest CT showed a space occupying lesion in the right hilum, multiple enlarged lymph nodes in the mediastinum, nodules in the left inferior lobe, thickening of bilateral interlobular septa and thickening of the right lobular septa and thickening of the right, lobular fissure, pneumonia (Figure 1). After 12 days of hospitalization, mNGS identified 320 sequence reads corresponding to *Mycobacterium* in the lung tissue samples, among which 262 corresponded to *M. avium*, accounting for 81.2% (262/320) (Table 1). Therefore, this patient was *M. avium* positive, and the antibiotic regimen was changed. Clarithromycin (0.5 g), rifampicin (0.45 g), levofloxacin (0.5 g), and ethambutol (1.25 g) were given once daily, and linezolid (0.2 g) was given twice a day as recommended by guidelines (**Figure 6A**). However, the patient did not respond well to the antibiotic drugs, and the anti-inflammatory drug methylprednisolone was given. In the following days of treatment, the patient still had recurrent fever with temperatures as high as 40°C, which could not subside on its own. Thereafter, two more antibiotics, moxifloxacin and etimicin, were added, and after 7 days of combined treatment (7 anti-NTM antibiotics) the fever remitted. The patient's temperature returned to normal after continued treatment with this regimen for more than 20 days, and his condition improved (**Figure 6A**).

**Table 1:** Summary of results from traditional tests and mNGS.

**Patient**	**Sample**	**Traditional microbiological testing results**	**mNGS results**
Case 1	Lung tissue	Negative	*M. avium*
Case 2	BALF	Negative	*M. avium*
Case 3	Blood	NTM	*M. wolinskyi; M. Goodii; M. Smegmatis M. mageritense*

**Figure 2:**
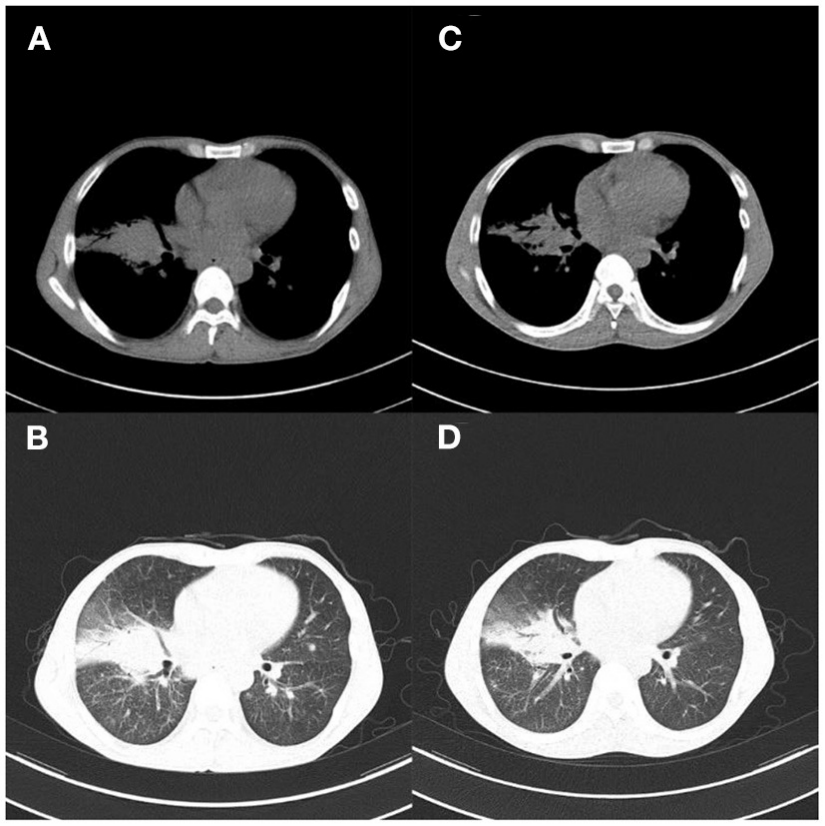
Case 1. **(A,B)** Chest CT 1 month after hospitalization showed improved pulmonary inflammation and reduced lesions; **(C,D)** Chest CT reexamination 38 days after admission showed more absorption of lesions, and significantly improved lung inflammation.

**Figure 3:**
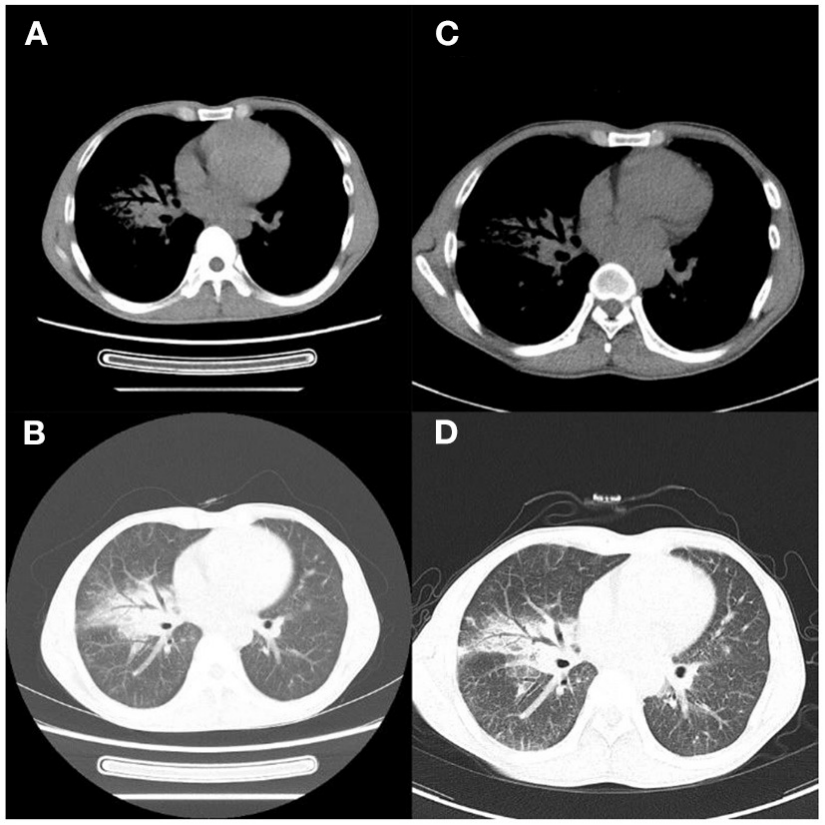
Case 1. **(A,B)** Chest CT 4 months later showed improved lung lesions; **(C,D)** and scar formation in the right middle lobe bronchus 8 months later.

Chest CT scan 1 month after hospitalization showed reduced pulmonary inflammation and lesion (Figure 2). The patient's temperature gradually returned to normal, and his condition was stable. Chest CT on day 38 showed that more of the lesions were absorbed, and lung inflammation significantly improved (Figure 2). After a period of active treatment, the patient's temperature was normal, his general condition improved significantly, and he was discharged. The patient continued treatment with ethambutol, moxifloxacin, clarithromycin, rifampicin, and isoniazid after discharge (**Figure 6A**). On follow-up 4 months later, the patient was in good general condition, with good sleep and no significant change in body weight, and his lung lesions improved on chest CT (Figure 3). This patient came back for reexamination eight months later, and his CT showed scar formation in the right middle lobe bronchus, and a few thin secretions were seen during bronchoscopy (Figure 3). These results indicate that the treatment was effective.

#### Case 2

**Figure 4:**
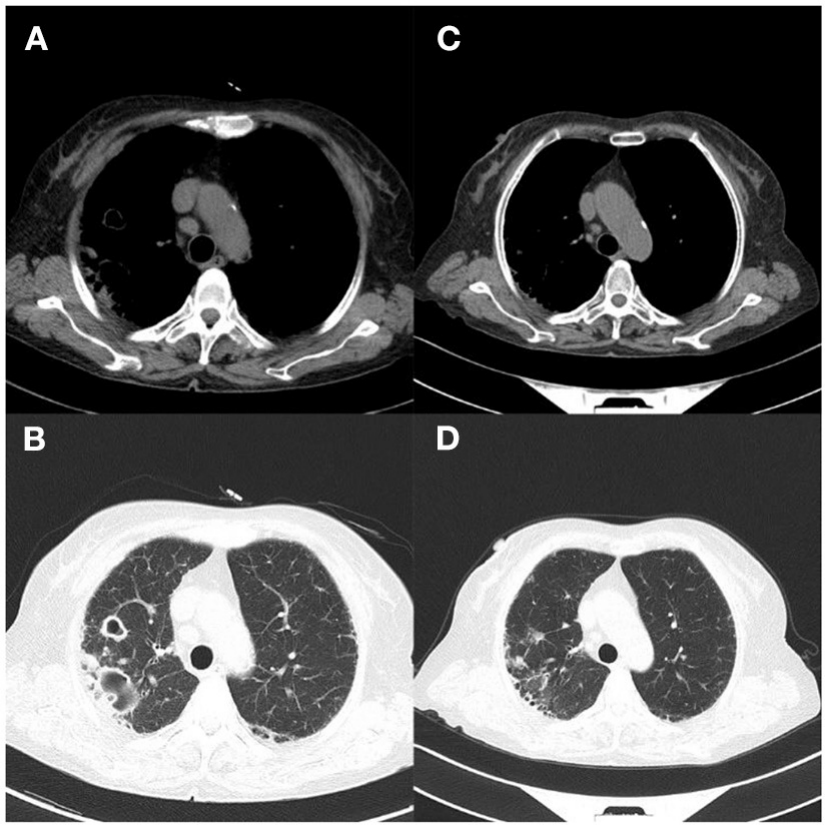
Case 2. **(A,B)** Lung CT on the day of admission showed interstitial inflammation in both lungs and cavity formation in the upper lobe of the right lung, localized emphysema of both lungs, mediastinal lymph node enlargement, and bilateral localized pleural thickening; **(C,D)** 1 month later, CT examination showed that the double pneumonia was slightly more advanced than before.

A 63-year-old female was admitted to our hospital complaining of intermittent multiple joint swelling and pain for more than 30 years, nausea for 7 months, and cough for 6 months. She had rheumatoid arthritis, interstitial pneumonia, osteoporosis, and systemic lupus erythematosus. The patient was treated with the immunosuppressive agent hydroxychloroquine during hospitalization and tripterygium wilfordii and pavlin immunosuppressive agents outside the hospital. She was previously treated at her local hospital using antibiotics, phlegm and cough relieving drugs, and IV fluids, which were not effective, and the patient's cough worsened 20 days before coming in. Laboratory tests on the first day of admission showed red blood cell count 3.59 × 10^12^ cells/L (reference range: 3.8–5.1), hemoglobin 103.0 g/L (reference range: 115–150), lymphocyte count 1.01 × 10^9^ cells/L (reference range: 1.1–3.2), monocyte count 0.73 × 10^9^ cells/L (reference range: 0.1–0.6), C-reactive protein 59.25 mg/L (reference range: 0–10), erythrocyte sedimentation rate 72 mm/h (reference range: 0–20). Acid-fast staining (sputum) was positive 4 and 6 days after admission. No bacterial growth was observed after 2 days of culture with common bacteria. Lung CT showed interstitial inflammation in both lungs, a cavitary lesion in the upper lobe of the right lung, localized emphysema in both lungs, mediastinal lymph node enlargement, and bilateral localized pleural thickening (Figure 4).

During hospitalization, her rheumatoid arthritis was treated with bitter melon extract and hydroprednisone, and her osteoporosis with calcitriol. Five days after admission, mNGS revealed infection with MAC; 2121 sequences corresponding to *Mycobacterium* were detected, among which 362 (17.1%) were MAC (Table 1). The antibiotic medication regimen was adjusted to ethambutol (0.75 g) and rifampin (0.45 g) every day before breakfast, levofloxacin (0.50 g) once every night, and clarithromycin (0.50 g) twice a day. After treatment with this antibiotic regimen, the patient's symptoms improved significantly, and she was discharged from the hospital (**Figure 6B**). CT examination on her one-month follow-up showed a slight progression of double pneumonia since being discharged from the hospital (Figure 4).

#### Case 3

**Figure 5:**
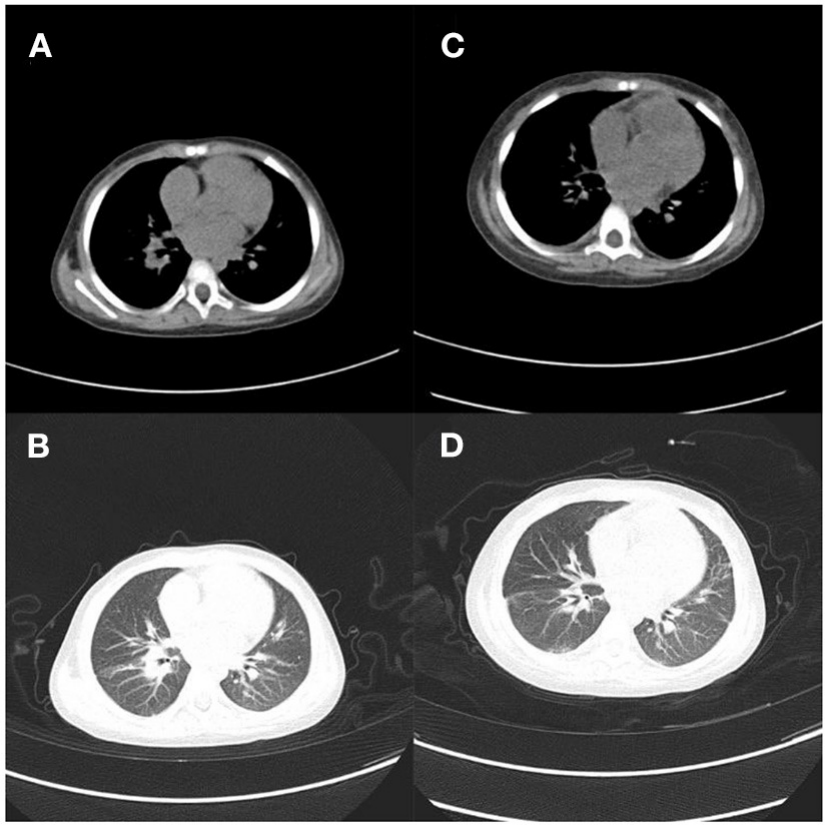
Case 3. **(A,B)** Lung CT on day 1 showed mild lung infection, multiple nodules and thickening of the anterior mediastinal soft tissue; **(C,D)** Chest CT showed worsened infection in both lungs, multiple small nodules, and new bilateral pleural effusion 2 months later.

**Figure 6:**
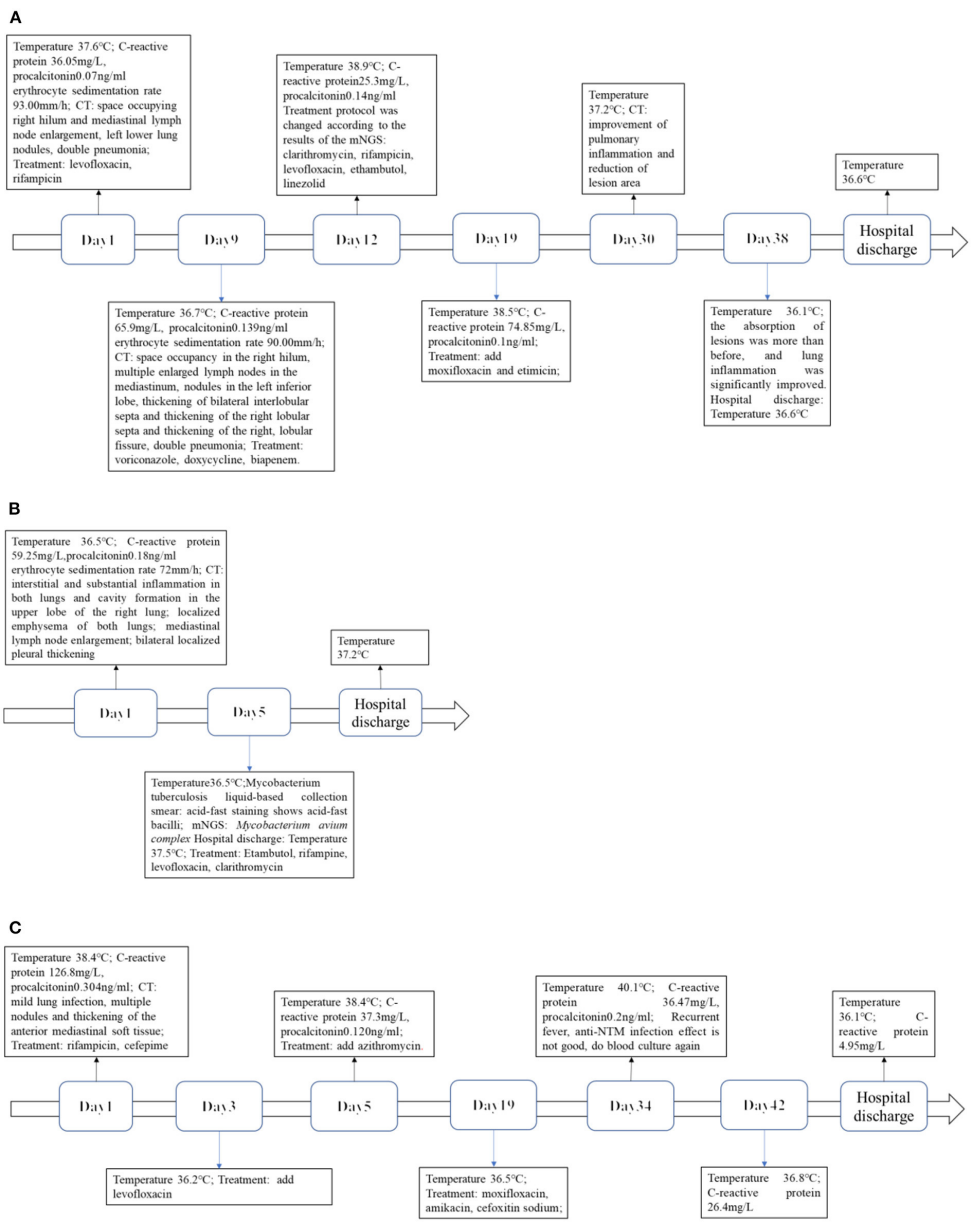
**(A)** Case 1. Timeline of infection progression. **(B)** Case 2. Timeline of infection progression. **(C)** Case 3. Timeline of infection progression.

A 2-year-old girl was hospitalized for more than 10 months for acute lymphoblastic leukemia and had a fever for 1 day. Her medication history includes a chemotherapy regimen comprising of vindesine, daunorubicin, pegaspargase, and prednisone, followed by cyclophosphamide, cytarabine, and 6-mercaptopurine chemotherapy, and vindesine was used as the maintenance therapy. During hospitalization, chemotherapy was halted due to bone marrow myelosuppression. Laboratory tests on the day of admission showed red blood cell count 3.76 × 10^12^ cells/L (reference range: 3.8–5.1), hemoglobin 102.1 g/L (reference range: 115–150), white blood cell count 0.77 × 10^9^cells/L (reference range: 3.5–9.5), neutrophil count 0.03 × 10^9^cells/L (reference range: 40–75), C-reactive protein 126.8mg/L (reference range: 0–5), and procalcitonin 0.304 ng /ml (reference range: 0–0.046). Mycobacterium culture was positive twice in the past hospitalization. Mycobacterial cultures were positive on days 11, 20, and 26 of admission. There was no common bacterial growth in blood culture for 5 days after admission 22 days. mNGS identified *Mycobacterium wolinskyi, Mycobacterium goodii, Mycobacterium smegmatis* and *Mycolicibacterium mageritense* and BK virus. The results of mNGS and blood culture showed NTM infection (Table 1). Lung CT showed mild infection with multiple nodules and thickening of the anterior mediastinum (Figure 5). This patient was eventually diagnosed with disseminated NTM disease, infective endocarditis, acute lymphoblastic leukemia, hypoproteinemia, cardiac insufficiency, multiple serous cavity effusion, and liver injury. Intermittent fever and increased inflammatory markers persisted even after combined treatment with rifamycin, cefepime, levofloxacin, and azithromycin (Figure 6C). The patient's temperature returned to normal after being given a low dose of dexamethasone. Because her overall response to the treatment was not ideal, the regimen was changed to a combination of amikacin, moxifloxacin, and cefoxitin, after which the patient's body temperature became stable, suggesting that this antibiotic regimen was effective (Figure 6C). Follow-up 2 months later showed that the lesions had shrunk, but she still had a recurrent fever, and blood cultures remained positive. Chest CT showed infection in both lungs and multiple small nodules that were more severe than on the last scan, and a new bilateral pleural effusion (Figure 5). Clarithromycin, cefoxitin, and amikacin were added to her regimen based on our experience and treatment guidelines. The patient was discharged because her vital signs were stable. One month later, the patient underwent surgery to remove tricuspid valve vegetations and replace the tricuspid valve. Unfortunately, this patient eventually died of severe postoperative infection and multiple organ failure.

## Discussion

In this paper, we report three cases of NTM-LD diagnosed using mNGS. The preferred treatment for NTM infections is drug therapy, but treatment varies depending on the NTM causing the infection. However, treatment outcome is usually poor because of the increasing resistance of NTM to many drugs. The recommended treatment for MAC is rifampicin, ethambutol, and macrolides for 18–24 months. Aminoglycosides, such as amikacin or streptomycin, may be supplemented in severe cases for the first 3–6 months (8). If left untreated, MAC lung disease (MAC-LD) can progress and lead to extensive lung destruction and respiratory failure. Chronic MAC-LD, if left untreated, has a 33.3% mortality rate and a 22.2% 5-year mortality rate (9).

Of the three NTM-LD cases reported in this study, two were refractory. Case 1 was a case of refractory NTM with granulomatous lesions on pathological studies and recurrent fever during treatment with isoniazid, rifampicin, ethambutol, and clarithromycin, with an additional 6 months of moxifloxacin that may have improved his prognosis (10, 11). At present, there is no universal standard that offers a best treatment for refractory MAC-LD. However, the use of clofazimine and bedaquiline has been reported to increase negative rates in cultures (12). Bedaquiline, developed for the treatment of multidrug-resistant tuberculosis, has antimicrobial activity against NTM species, including *M. abscessus* and MAC (13). Bedaquiline can be supplemented to treat disseminated NTM infections in immunocompromised patients, such as those with HIV/AIDS (14). The addition of amikacin liposome inhalation suspension in the treatment of refractory MAC-LD significantly increases the probability of sputum culture conversion while reducing the risk of systemic toxicity compared with intravenous aminoglycosides (15).

Case 2 was reexamined a month after discharge, and lung CT showed progression of inflammation and the cavitary lesion compared to before, which we believe were due to insufficient treatment time or drug resistance. Pulmonary cavitation is a hallmark of tuberculosis and is associated with antibiotic resistance (16). The supply and penetration of drugs into the lesion was reduced because the vasculature within the cavity was destroyed (17). A study reported a higher treatment success rate when aminoglycosides were used for more than 3 months in patients with MAC-LD (18). Parenteral amikacin or streptomycin is recommended by guidelines as the initial treatment regimen for patients with MAC-LD with cavitary lesions and macrolide resistance (2).

Case 3 is a young child with chemotherapy-induced immunosuppression. This case is representative of increased susceptibility to NTM in immunocompromised patients. For instance, *M. wolinskyi* infections are uncommon but reported to be associated with postsurgical wound infections, sternal osteomyelitis, and infective endocarditis. Amikacin, an aminoglycoside, may be a good first-choice treatment for *M. wolinskyi* infections, which have been reported to be sensitive to cefoxitin, clarithromycin and, quinolones. This case was particularly difficult to treat because of her impaired immune system which led to a poor prognosis and is usually the case for patients undergoing treatment for hematologic malignancies.

Timely detection of NTM was achieved with mNGS, and these three cases were accurately diagnosed and effectively treated. More and more research stress the dangers of the increasing incidence of NTM infections, and the emergence of mNGS will become an effective tool for their diagnosis. mNGS overcome the limitations of traditional detection methods by simultaneously identifying a variety of pathogens. However, the extensive application of mNGS as a first-line diagnostic measure is limited by their high equipment and operational costs, and in most cases, it is only used as a supplement to conventional detection methods. It would be of great benefit to use mNGS as a first-line test to diagnose difficult or complex infectious diseases, especially in immunocompromised patients. mNGS is a revolutionary test method (19) that holds great potential and is receiving increasing support from more and more research for its application.

## Data availability statement

The datasets presented in this study can be found in online repositories. The names of the repository/repositories and accession number(s) can be found below: https://db.cngb.org/, Project Number: CNP0003516.

## Ethics statement

The studies involving human participants were reviewed and approved by The First Affiliated Hospital of Zhengzhou University. Ethics approval number is 2019-KY-330. Written informed consent to participate in this study was provided by the participants' legal guardian/next of kin. Written informed consent was obtained from the individual(s), and minor(s)' legal guardian/next of kin, for the publication of any potentially identifiable images or data included in this article.

## Author contributions

ZY, YL, and XM analyzed and interpreted patient data. YL and XM wrote the manuscript. JC and HW revised the article. HW and YL analyzed the genomics data. All authors have read and approved the final manuscript.

## Funding

This research was equally funded and supported by Chinese National Science and Technology Major Project 2018ZX10305410, Henan Province Medical Science and Technique Project Grant 2018020001, and Henan Province Postdoctoral Research Grant 001801005.

## Conflict of interest

The authors declare that the research was conducted in the absence of any commercial or financial relationships that could be construed as a potential conflict of interest.

## Publisher's note

All claims expressed in this article are solely those of the authors and do not necessarily represent those of their affiliated organizations, or those of the publisher, the editors and the reviewers. Any product that may be evaluated in this article, or claim that may be made by its manufacturer, is not guaranteed or endorsed by the publisher.

## References

[1] Ahmed I Tiberi S Farooqi J Jabeen K Yeboah-Manu D Migliori GB. Non-tuberculous mycobacterial infections—a neglected and emerging problem. Int J Infect Dis IJID Off Publ Int Soc Infect Dis. (2020) 92s:S46–s50. 10.1016/j.ijid.2020.02.022 32114200

[2] Daley CL Iaccarino JM Lange C Cambau E Wallace RJ Andrejak C. Treatment of nontuberculous mycobacterial pulmonary disease: an official ATS/ERS/ESCMID/IDSA clinical practice guideline. Eur Resp J. (2020) 56:e1–e36. 10.1183/13993003.00535-2020 32628747 PMC7768748

[3] Van Ingen J Aksamit T Andrejak C Böttger EC Cambau E Daley CL. Treatment outcome definitions in nontuberculous mycobacterial pulmonary disease: an NTM-NET consensus statement. Eur Resp J. (2018) 51:1800170. 10.1183/13993003.00170-2018 29567726 PMC6660914

[4] Gopalaswamy R Shanmugam S Mondal R Subbian S. Of tuberculosis and non-tuberculous mycobacterial infections—a comparative analysis of epidemiology, diagnosis and treatment. J Biomed Sci. (2020) 27:74. 10.1186/s12929-020-00667-6 32552732 PMC7297667

[5] Cassidy PM Hedberg K Saulson A Mcnelly E Winthrop KL. Nontuberculous mycobacterial disease prevalence and risk factors: a changing epidemiology. Clin Infect Dis. (2009) 49:e124–9. 10.1086/648443 19911942

[6] Kalantar K L Carvalho T De Bourcy C F A Dimitrov B Dingle G Egger R. IDseq-An open source cloud-based pipeline and analysis service for metagenomic pathogen detection and monitoring. GigaScience. (2020) 9:giaa111. 10.1093/gigascience/giaa111 33057676 PMC7566497

[7] Jiao M Ma X Li Y Wang H Liu Y Guo W. et al. Metagenomic next-generation sequencing provides prognostic warning by identifying mixed infections in nocardiosis. Front Cell Infection Microbiol. (2022) 12:894678. 10.3389/fcimb.2022.894678 36118026 PMC9471186

[8] Van Ingen J Boeree M Van Soolingen D Mouton JJDRUR Antimicrobial CI. Chemotherapy A. Resistance mechanisms and drug susceptibility testing of nontuberculous mycobacteria. Drug Resist Updat. (2012) 15:149–61. 10.1016/j.drup.2012.04.001 22525524

[9] Diel R Nienhaus A Ringshausen F C Richter E Welte T Rabe K F. Microbiologic outcome of interventions against mycobacterium avium complex pulmonary disease: a systematic review. Chest. (2018) 153:888–921. 10.1016/j.chest.2018.01.024 29410162

[10] Hopewell P Cynamon M Starke J Iseman M O'Brien R. Evaluation of new anti-infective drugs for the treatment and prevention of infections caused by the Mycobacterium avium complex. Infect Dis Soc Am Food Drug Admin. (1992) 15:S296–306. 10.1093/clind/15.Supplement_1.S296 1477245

[11] Koh W J Hong G Kim S Y Jeong B H Park H Y Jeon K. Treatment of refractory Mycobacterium avium complex lung disease with a moxifloxacin-containing regimen. Antimicrob Agents Chemother. (2013) 57:2281–5. 10.1128/AAC.02281-12 23478956 PMC3632919

[12] Daley CL Winthrop KL. Mycobacterium avium complex: addressing gaps in diagnosis and management. J Infect Dis. (2020) 222:S199-211. 10.1093/infdis/jiaa354 32814943 PMC7566660

[13] Cowman S van Ingen J Griffith DE Loebinger MR. Non-tuberculous mycobacterial pulmonary disease. Eur Resp J. (2019) 54. 10.1183/13993003.00250-2019 31221809

[14] Gil E Sweeney N Barrett V Morris-Jones S Miller RF Johnston VJ. Bedaquiline as treatment for disseminated nontuberculous mycobacteria infection in 2 patients co-infected with HIV. Emerg Infect Dis. (2021) 27:944–8. 10.3201/eid2703.202359 33622490 PMC7920675

[15] Hirley M. Amikacin liposome inhalation suspension: a review in Mycobacterium avium complex lung disease. Drugs. (2019) 79:555–62. 10.1007/s40265-019-01095-z 30877642 PMC6445814

[16] Ong CW Elkington PT Friedland JS. Tuberculosis, pulmonary cavitation, and matrix metalloproteinases. Am J Respir Crit Care Med. (2014) 190:9–18. 10.1164/rccm.201311-2106PP 24713029 PMC4226026

[17] Dartois V. The path of anti-tuberculosis drugs: from blood to lesions to mycobacterial cells. Nat Rev Microbiol. (2014) 12:159–67. 10.1038/nrmicro3200 24487820 PMC4341982

[18] Kim O Kwon B Han M Koh Y Kim W Song J. Association between duration of aminoglycoside treatment and outcome of cavitary mycobacterium avium complex lung disease. Clin Infect Dis. (2019) 68:1870–6. 10.1093/cid/ciy804 30239615

[19] Li N Cai Q Miao Q Song Z Fang Y Hu B. High-throughput metagenomics for identification of pathogens in the clinical settings. Small Methods. (2021) 5:2000792. 10.1002/smtd.202000792 33614906 PMC7883231

